# Catalytic hydrogenolysis of lignin to phenolic monomers over Ru supported N,S-co-doped biochar: The importance of doping atmosphere

**DOI:** 10.3389/fchem.2022.1022779

**Published:** 2022-09-13

**Authors:** Wenran Gao, Ke Wang, Yishuang Wu, Xun Zhu, Yinlong Wu, Shoujun Zhang, Bin Li, Yong Huang, Shu Zhang, Hong Zhang

**Affiliations:** ^1^ Joint International Research Laboratory of Biomass Energy and Materials, Jiangsu Co-Innovation Center of Efficient Processing and Utilization of Forest Resources, College of Materials Science and Engineering, Nanjing Forestry University, Nanjing, Jiangsu, China; ^2^ Department of Chemistry, Shantou University Medical College, Shantou, Guangdong, China; ^3^ Hefei Debo Bioenergy Science & Technology Co., Ltd., Hefei, Anhui, China; ^4^ School of Energy and Power Engineering, Jiangsu University, Zhenjiang, China

**Keywords:** lignin, phenolic monomers, N,S-co-doping, biochar, hydrogenolysis

## Abstract

Doping of heteroatoms into carbon materials is a popular method to modify their physicochemical structures and has been widely used in the fields of energy conversion and storage. This study aims to investigate the effect of doping atmosphere on the catalytic performance of nitrogen and sulfur co-doped biochar supported Ru in the production of phenolic monomers from lignin hydrogenolysis. The results showed that the catalyst prepared under CO_2_ atmosphere (Ru@CNS-CO_2_) was able to produce phenolic monomers from corncob lignin with a yield up to 36.41 wt%, which was significantly higher than that from the run over N_2_-prepared catalyst (Ru@CNS-N_2_). The characterization of the catalysts demonstrated that the CNS-CO_2_ support had a larger specific surface area, richer C=S and C-S groups, and higher oxygen content than CNS-N_2_, resulting in finer Ru particles and more Ru^0^ content on the CNS-CO_2_ support. The Ru@CNS-CO_2_ catalyst exhibited high activity in hydrogenation and fragmentation of β-O-4 linkages.

## Introduction

With the exhaustion of fossil energy and the emission of carbon dioxide during its utilization, biomass conversion has become a hot topic because of its renewability and carbon neutrality or even negativity. Lignin, which accounts for 15–30 wt% of biomass, consists of coniferyl alcohol (G), sinapyl alcohol (S), and *p*-coumaryl alcohol (H) phenylpropane units connected by C-O/C bonds, and is usually considered to be the largest source of renewable aromatic-containing compounds in nature (Zakzeski et al., 2010; Shuai et al., 2016). The main method for lignin valorization is to break the linkages among structural units to form small molecular phenolic compounds, which has great potentials in important platforms to produce fuels, phenolic resin, and other chemicals (Ragauskas et al., 2014; Schutyser et al., 2018; Zevallos Torres et al., 2020). Lignin depolymerization into chemicals and fuels, however, remains a challenge due to the stability and complexity of its structure.

Many strategies, such as catalytic pyrolysis, oxidation, hydrogenolysis, have been developed to break the C-O/C bonds among structural units (Li et al., 2015; Chen et al., 2021). Among them, catalytic hydrogenolysis is considered as one of the most promising methods because of the high selectivity to break specific bonds (Ye et al., 2021). Transition metals loaded on various supports are commonly employed as effective catalysts for the hydrogenolysis of lignin, which has led to two research directions: 1) exploration of the types of metals and/or the synergistic effect of multiple metals. For example, Cheng et al. compared the effects of Ni/C, Cu/C, and NiCu/C catalysts on depolymerization of organosolv poplar lignin, and NiCu/C was found to have the best catalytic performance among them (Cheng et al., 2020). Also, Li et al. investigated the activity of M/NiAl_2_O_4_ (M = Pt, Pd, Ru) catalysts in lignin hydrogenolysis, and Ru/NiAl_2_O_4_ showed the highest activity due to the rapid recovery of active sites (Li et al., 2021). 2) modification of the structures of supports to adjust the interactions between metals and supports, including the control of support composition and/or morphology, skeleton doping, and surface functionalization (van Deelen et al., 2019).

Among various support materials, biomass-derived biochar has attracted extensive attention because of the structural controllability, environmental friendliness, and low cost (Liu et al., 2015; Xiong et al., 2017). Since biochar is mainly composed of carbon atoms, doping heteroatoms (e.g., oxygen, nitrogen, sulfur, phosphorus, etc.) into biochar structure (including skeleton doping and surface functionalization) will inevitably affect the electron cloud density distribution of biochar, thus leading to change in interactions between metals and biochar. Recently, Luo et al. used nitrogen-doped biochar to load Ru particles as a catalyst and a high yield of phenolic monomer with 31.2 wt% was obtained by cornstalk lignin hydrogenolysis at 260°C (Luo et al., 2022). They claimed that the doping of nitrogen led to the formation of micro-mesoporous structure and promoted the electron transfer between the loaded Ru and biochar. Our previous study also investigated lignin hydrogenolysis over Ru supported on various (non)-functionalized graphitized carbon nanotubes (CNT, CNT-OH, CNT-COOH, and CNT-NH_2_), and Ru@CNT-NH_2_ provided the highest yield of phenolic monomers due to the prominent partial hydrogenation of C (sp^2^)-O/C bonds to C (sp^3^)-O/C bonds in lignin (Wu et al., 2021).

It should be noted that the doping of heteroatoms into biochar was usually conducted under an inert atmosphere (e.g., N_2_ and Ar), indicating that the insertion of heteroatoms into biochar was highly temperature-dependent. This often results in an inefficient doping process and non-uniform distribution of heteroatoms, which further affects the distribution of metals. It can be known from gasification of coal/biochar that carbon is easy to react with some active atmospheres (such as O_2_, CO_2_, and H_2_O), thus forming defects in carbon skeleton. Particularly for CO_2_, it can selectively react with the small aromatic rings in the carbon structure (Zhang et al., 2022). Inspired by this, doping of heteroatoms under an active atmosphere is expected to create more sites for doping and help the insertion of heteroatoms into carbon skeleton.

In this study, nitrogen and sulfur co-doped biochars in N_2_ and CO_2_ atmospheres were prepared as supports. After loading Ru onto the prepared supports, various characterizations were conducted to understand the differences in the physiochemical structures of the catalysts. Finally, catalytic hydrogenolysis of a technical lignin was performed to investigate the effect of doping atmosphere, with an emphasis on the production of phenolic monomers.

## Experiments

### Materials

Technical lignin (40–200 mesh) was purchased from Shandong Longlive Biotechnology Co., Ltd., China. Ruthenium chloride hydrate (RuCl_3_•xH_2_O) with Ru content 35–42% was purchased from Aladdin Technical Corporation, China. Methanol (>99.5%) was purchased from Nanjing Chemical Reagent Co., Ltd., China. Thiourea (≥99.0%) was purchased from Sinopharm Chemical Reagent Co., Ltd. Deionized water was used throughout the experiment.

### Catalysts preparation

The biochar support was obtained from poplar pyrolysis under a N_2_ atmosphere at 450°C for 3 h. Nitrogen and sulfur co-doped supports were prepared by carbonizing a mixture of char and thiourea at a 1:2 mass ratio under a N_2_ or CO_2_ atmosphere at 800°C for 1 h, which were noted as CNS-N_2_ and CNS-CO_2_ later. To understand the effect of nitrogen and sulfur co-doping, a sole biochar sample was also carbonized at 800°C under N_2_ atmosphere to prepare a undoped support (marked as C). Then, the supports were volume-impregnated by a mixed solution of RuCl_3_•xH_2_O dissolved by ethanol and water (1:1 volume ratio) to load Ru, the amount of which was precalculated of 5 wt%. The dried samples were reduced at 400°C for 3 h with a mixed atmosphere flow of 5% H_2_ and 95% Ar (total 1 L/min) to finally obtain the Ru@C, Ru@CNS-N_2_ and Ru@CNS-CO_2_ catalysts⁰.

### Catalytic performance tests

For each hydrogenolysis reaction, 1 g of dried technical lignin, 0.2 g catalyst and 30 ml methanol were put into a 100 ml mechanical stirring autoclave. Residual air in the sealed autoclave was purged with nitrogen and hydrogen for five times successively, and then 1 MPa H_2_ was pressurized. The reaction was maintained at 250°C for 2 h. After that, the reactor was cooled down to room temperature and the liquid products were collected for qualitative and quantitative analysis.

The obtained organic soluble oil was analyzed by gas chromatography/mass spectrometry (GC/MS) equipped with column Agilent J&W VF-1701ms (30 m × 250 μm × 0.5 μ m). The column was heated to 200°C at the rate of 5°C/min from the initial temperature of 40°C, and then raised to 280°C at the rate of 10°C/min and held for 3 min. The external standard sample concentrations of five concentrations were used for the standard curve, and then the concentration of the compound was calculated according to the peak area. The yield of phenolic monomer was calculated according to the following formula:

$$Y_{monomer}=\frac{C_{monomer}\times V}{1000\times m_{lignin}}$$ (1)

where *Y*_monomer_ (%) was the monomer yield based on the weight of technical lignin; *C*_monomer_ (g/L) represented the concentration calculated by the peak area; V (ml) was the total volume of liquid sample taken for GC/MS analysis; and m_lignin_ (g) was the mass of technical lignin.

### Catalyst characterization

Elemental analysis to determine the contents of C, S, N and H of ash-free base of the catalysts was conducted on a Vario ELIII elemental analyzer (Elementar Company, Gernany). The Brunauer–Emmett–Teller method (BET) with a nitrogen adsorption/desorption isotherm was conducted on BSD-PM4 analyzer from BSD INSTRUMENT (Beijing) to obtain the specific surface area. The degassing temperature was 300°C, and the degassing time was 510 min. The prepared catalysts were also analyzed by FTIR (Bruker Vertex 80V, Germany) using the potassium bromide pellet technique, in which a KBr:char ratio of 100:1 (w:w) was used. The FTIR spectral region was from 400 to 4,000 cm^−1^. The carbon structures of the catalysts were measured by a Raman technique spectrometer (ThermoFisher DXR532). The excitation laser wavelength used in the equipment was 780 nm, while the laser power was 24 mW. Based on previous study (Zhang et al., 2011; Wang et al., 2017), the original Raman spectra were curve-fitted into 10 Gaussian bands, in which the ratio of D to (G_R_ + V_L_ + V_R_) band areas would suggest the ratio of the large to small aromatic ring systems. Temperature-programmed desorption (TPD) was conducted on a PCA-1200 BUILDER chemical adsorption analyzer (China) to analyze the surface acid sites. The samples were first saturated using 10 vol% NH_3_ (30 ml/min) at 50°C for 30 min. After purging by N_2_ (30 ml/min) at 50°C for 1 h, the NH_3_-TPD was conducted by raising temperature from 50 to 650°C at a rate of 10°C/min and keeping it at 650°C for 1 h. X-ray photoelectron spectroscopy (XPS) was measured to investigate the chemical states of elements, such as Ru, C, N and S, relying on AXIS UltraDLD (Shimadzu, Japan) instrument equipped with Al Kα radiation (150 W), whose wide pass energy was 160 eV and narrow pass energy was 40 eV. Before testing, pure silica was mixed up with the catalysts as an internal standard to calibrate the binding energies. High-resolution transmission electron microscopy (HRTEM) was carried out to analyze the Ru particle size and observe the dispersion, of which the average particle diameter (*d*) was calculated according to the following formula:

$$d=\frac{\sum n_{i}d_{i}^{3}}{\sum n_{i}d_{i}^{2}}$$ (2)

where *n*_*i*_ was the count of particles and *d*_*i*_ was each characteristic diameter of particles.

## Results and discussion

### Characteristics of Ru@C, Ru@CNS-N_2_ and Ru@CNS-CO_2_

**FIGURE 1:**
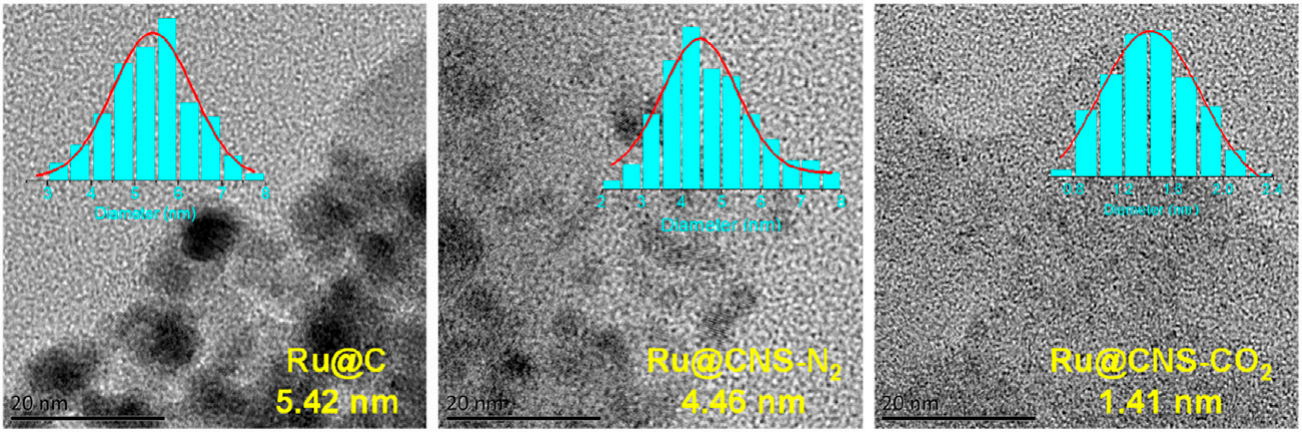
TEM images and histograms of particle size for the catalysts.

The three prepared catalysts were analyzed by various methods to understand the differences in their physicochemical structures caused by N,S co-doping and/or doping atmosphere. TEM analysis was conducted to measure the morphology and distribution of metal Ru particles on the three supports. It can be observed from Figure 1 that the Ru particles on the undoped char were agglomerated with an average size of 5.42 nm, while the Ru particles were well dispersed on the N,S-co-doped char with smaller sizes. Surprisingly, the particles size of Ru on CNS-CO_2_ was as small as 1.41 nm. This is consistent with the results of the study from Li et al., in which they found that the strong chemical interaction between metal and the doped sulfur atoms can greatly suppress the aggregation of metal species (Li et al., 2020). It has also been reported that smaller metal particles showed better catalytic performance during reactions (Che et al., 1989; Isaifan et al., 2013). Thus, Ru@CNS-CO_2_ was expected to have a good catalytic effect on lignin depolymerization.

**FIGURE 2:**
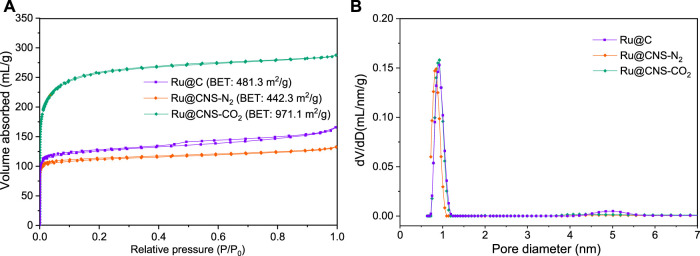
Nitrogen physical sorption isotherms **(A)** and pore size distribution **(B)** of the catalysts.

CO_2_ is a typical agent for activation of carbon material, and the specific surface area of the carbon material can be significantly improved. Figure 2A shows the nitrogen adsorption and desorption isotherms of the three catalysts, among which Ru@C belonged to a typical type IV isotherm, while Ru@CNS-N_2_ and Ru@CNS-CO_2_ were typical type I isotherm. The change of the isotherm type of the catalyst from type IV to type I indicated that the mesopores in the catalyst were consumed during the N,S-co-doping process, which was also proved by the pore size distribution shown in Figure 2B. Although the three catalysts were predominant in micropores, a small peak at around 5 nm for Ru@C could be observed. Compared with Ru@C, the specific surface area of Ru@CNS-N_2_ slightly reduced from 481.3 to 442.3 m^2^/g, indicating that the doping of nitrogen and sulfur into char under N_2_ atmosphere also consumed a portion of micropores. Conversely, the specific surface area of Ru@CNS-CO_2_ significantly increased to 971.1 m^2^/g due to the reaction of CO_2_ with carbon to generate micropores.

Table 1 lists the elemental composition of the three catalysts. The content of Ru was stabilized at 4.5–4.8 wt% for all prepared catalysts. Since the biochar support has experienced the temperature as high as 800°C, the carbon content in Ru@C reached 83.2 wt% and the oxygen content reduced to 11.3 wt%. After the biochar support was doped by nitrogen and sulfur, the carbon content in Ru@CNS-N_2_ reduced to 74.2 wt%. However, the reduction in carbon content did not necessarily mean that carbon was successfully replaced by heteroatoms, which could also be present as surface functional groups. Reasonably, the contents of nitrogen and sulfur, which were negligible in Ru@C, respectively increased to 6.3 and 2.8 wt% in Ru@CNS-N_2_. The doping process insignificantly affect the oxygen content. When the doping atmosphere changed from N_2_ to CO_2_, the carbon content obviously decreased to 58.5 wt%, mainly caused by the reaction between carbon and CO_2_. It is well known that CO_2_ atmosphere favors the formation of oxygen-containing functional groups on biochar surface under high temperature (Liu et al., 2020), which led to an oxygen content up to 25.0 wt% in Ru@CNS-CO_2_. It has been reported that the carbon atoms close to oxygen-containing functional groups had unique electron cloud densities and thus exhibited special interactions with Ru nanoparticles and/or lignin molecules (Li et al., 2019).

**TABLE 1:** Elemental and Raman analyses of the three catalysts.

Sample	C (%)	H (%)	N (%)	S (%)	Ru*^a^* (%)	O*^b^* (%)	I (G_R_ + V_L_ + V_R_)/I_D_
Ru@C	83.2	0.7	0.2	<0.1	4.5	11.3	0.81
Ru@CNS-N_2_	74.2	1.1	6.3	2.8	4.8	10.8	1.08
Ru@CNS-CO_2_	58.5	1.3	5.6	4.9	4.7	25.0	0.93

a Quantified by ICP-OES.

b By difference.

**FIGURE 3:**
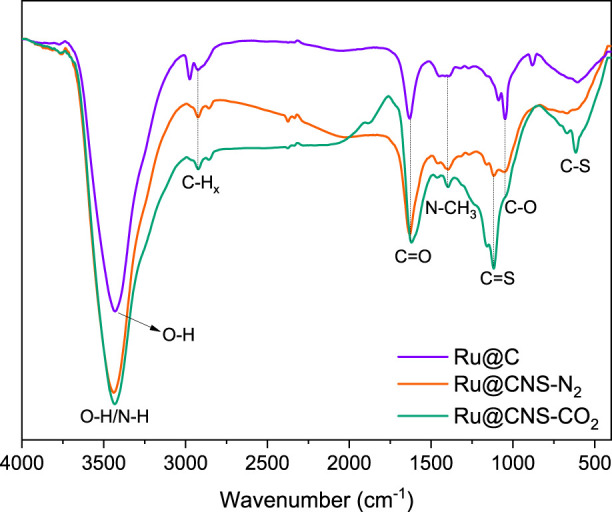
FTIR spectra of the three catalysts.

Figure 3 shows the FTIR spectra of the three catalysts. Compared with Ru@C, the band at around 3,435 cm^−1^ from Ru@CNS-CO_2_ and Ru@CNS-N_2_, which is assigned to O-H and/or N-H, became significantly higher because of the presence of N-H groups derived from nitrogen doping. The band at 1,620 cm^−1^ is assigned to C=O group. It is interesting to find that the doping of nitrogen and sulfur under both N_2_ and CO_2_ atmospheres favored the formation of C=O group. More importantly, the CO_2_ atmosphere was obviously beneficial to the doping of sulfur into the char carbon skeleton, since the signals of C=S and C-S groups from Ru@CNS-CO_2_ were much stronger than those from the others (Taheri-Torbati et al., 2017; Cui et al., 2018). Similar to oxygen doping, since sulfur has a larger electronegativity than carbon, the successful doping of sulfur into carbon skeleton can also cause changes in the electron cloud density of the char, which is expected to improve the catalytic performance of Ru@CNS-CO_2_.

**FIGURE 4:**
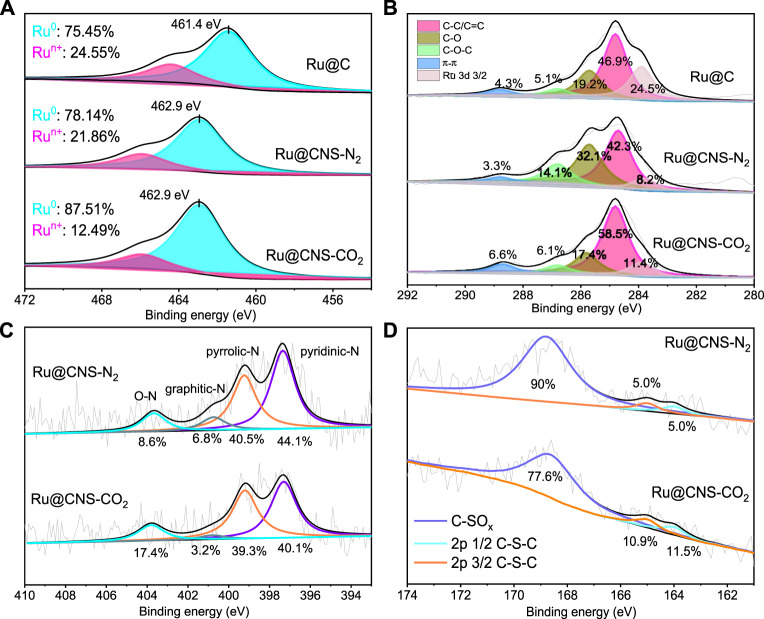
XPS spectra of **(A)** Ru 3p 3/2, **(B)** C 1s, **(C)** N 1s and **(D)** S 2p of the catalysts.

The chemical states of Ru, C, N, and S elements in the three catalysts were studied by XPS measurements. The XPS spectra of Ru 3p 3/2 can be deconvoluted into Ru^0^ and Ru^n+^ species, in which the Ru^0^ species had been recognized as the active sites for hydrogenolysis of lignin (Wu et al., 2021; Ding et al., 2022). As shown in Figure 4A, the content of Ru^0^ species slightly increased from 75.45% (Ru@C) to 78.14% (Ru@CNS-N_2_), suggesting that the co-doping of nitrogen and sulfur could facilitate the formation of Ru^0^ species. When the doping atmosphere changed from N_2_ to CO_2_, the content of Ru^0^ species in Ru@CNS-CO_2_ significantly increased to 87.51%. This can be explained by the better doping of sulfur into the carbon skeleton under CO_2_ atmosphere and the greater electronegativity of sulfur in comparison to carbon, thus promoting the electron transfer from CNS-CO_2_ support to Ru particles. The interactions between the doped chars and Ru particles were also demonstrated by the shift of the binding energy of metallic Ru (462.9 vs 461.4 eV). Figure 4B showed the XPS spectra of C 1s. Except for the overlapping peak of Ru 3d 3/2, the C 1s XPS spectra can be deconvoluted into C-C/C=C at 284.8 eV, C-O at 285.9 eV, C-O-C at 286.9 eV, and π-π bond at 289.4 eV. The content of C-C/C=C reduced from 46.9% (Ru@C) to 42.3% (Ru@CNS-N_2_), indicating that the doping of nitrogen and sulfur in N_2_ reduced the graphitization degree of the char to a certain degree. However, that number increased to 58.5% in the case of Ru@CNS-CO_2_, resulting from the selective consumption of small aromatic rings in carbon structure by CO_2_. This conclusion was also proved by the Raman analysis of the catalysts. The value of (G_R_ + V_L_ + V_R_)/D, which represents the ratio of the small to large aromatic ring systems in carbon materials (Huang et al., 2020), increased from 0.81 (Ru@C) to 1.08 (Ru@CNS-N_2_) and then decreased to 0.93 (Ru@CNS-CO_2_), as listed in Table 1. The N 1s XPS spectra, as exhibited in Figure 4C, were deconvoluted into pyridinic-N (398.6 eV), pyrrolic-N (400.5 eV), graphitic-N (402.0 eV), and nitrogen oxide (405 eV). The high contents of pyridinic-N and pyrrolic-N suggested the doping of nitrogen into carbon skeleton of char. Since the CO_2_ atmosphere would introduce oxygen-containing functional groups to the char surface, the content of the O-N species in Ru@CNS-CO_2_ was significantly higher than that in Ru@CNS-N_2_. Figure 4D shows the S 2p XPS spectra, which were deconvoluted into C-S-C species of S 2p 1/2 and S 2p 3/2 as well as C-SO_x_. The higher contents of C-S-C species in Ru@CNS-CO_2_ also indicated the successfully doping of sulfur into the carbon skeleton of char, which was in good agreement with the FTIR analysis.

**FIGURE 5:**
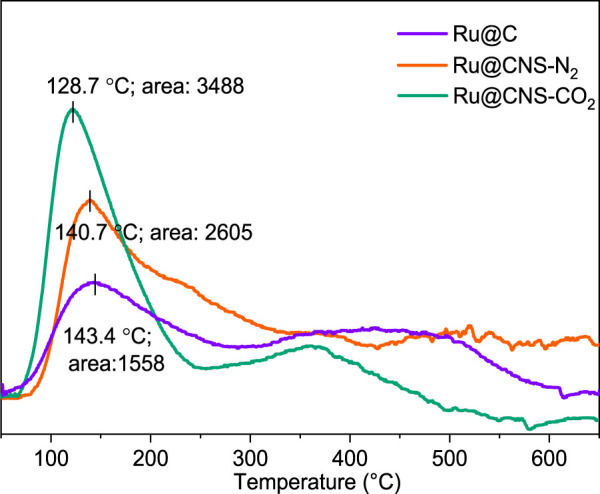
NH_3_-TPD patterns of the three catalysts.

The introduction of oxygen-, nitrogen-, and/or sulfur-containing functional groups into carbon materials significantly affects their acidity, NH_3_-TPD analysis was thus conducted to determine the acidic sites of the prepared catalysts. As shown in Figure 5, a small peak at 143.4°C with an area of 1,558 was found for Ru@C, suggesting the presence of very few weakly acidic sites. When the nitrogen and sulfur were doped into the char, the peak was slightly shifted to 140.7°C with an area of 2,605 (Ru@CNS-N_2_) and 128.7°C with an area of 3,488 (Ru@CNS-CO_2_), indicating that the acidity of the catalyst became weaker but the acid sites were more abundant, particularly under the CO_2_ atmosphere.

### Production of phenolic monomers from lignin depolymerization over the three catalysts

**FIGURE 6:**
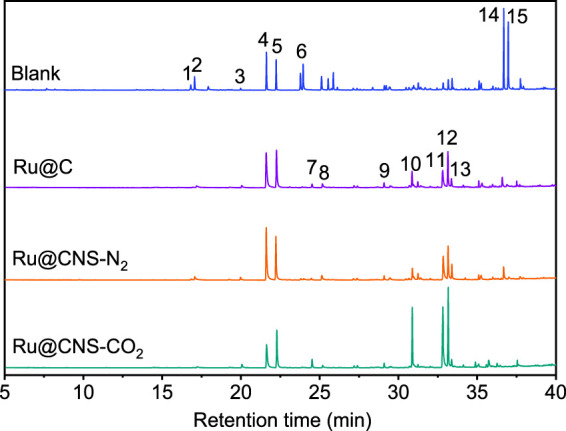
TICs of the products from lignin depolymerization over different catalysts.

**FIGURE 7:**
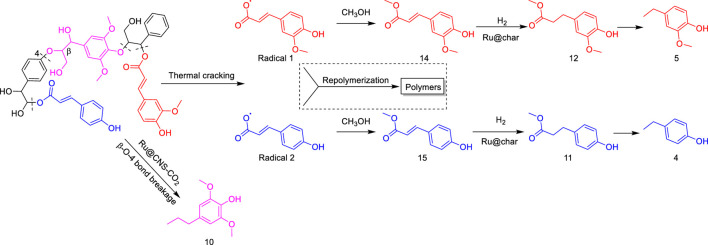
Schematic diagram of lignin depolymerization to the main compounds.

On the basis of understanding the properties of the three catalysts, a technical lignin derived from corncob was selected to test their catalytic performances on the production of phenolic monomers. The liquid products from the lignin depolymerization were qualitatively and quantitatively analyzed by GC/MS, and the total ion chromatograms (TICs) of the products as well as the yield of each compound are shown in Figure 6 and Table 2, respectively. For comparison, a run without catalyst was also conducted, in which the total yield of all identified phenolic monomers was only 10.25 wt% (entry 16, Table 2). The most abundant compounds from the blank run, compounds **14** (methyl ferulate, 2.38 wt%) and **15** (methyl coumarate, 2.74 wt%), were not the products from the typical β-O-4 subunits. When the non-doped catalyst (Ru@C) was employed, the richest product shifted to compound **12** (methyl 3-(4-hydroxy-3-methoxyphenyl)propanoate, 5.14 wt%), followed by compounds **5** (4-ethyl-2-methoxyphenol, 4.47 wt%) and **4** (4-ethylphenol, 4.03 wt%). It should be noted that compound **12** was a hydrogenation product of compound **14**. Based on our previous study, the saturation of the double bond (Cβ = C_γ_) in compounds **14** and **15** reduced the bond dissociation energies of C_β_-C_γ_, thus contributing to the production of compounds **five** and **four** by decarboxylation, respectively (Wu et al., 2021). With the help of Ru@C, the total yield of phenolic monomers of corncob lignin depolymerization increased to 20.47 wt%. Repolymerization of lignin-derived intermediates to macromolecules has been recognized as common reactions during lignin conversion, which led to the low yield of total phenolic monomers from the blank run (Deuss et al., 2015). Since the compounds **12**, **4**, and **five** could be derived from the products **14** and **15**, the improved yield of total phenolic monomers from the Ru@C run meant that the catalyst not only facilitated the depolymerization of lignin to compounds **14** and **15** but also hindered their repolymerization. Unfortunately, the yield of total phenolic monomers and the main composition of the lignin oil from the run over Ru@CNS-N_2_ were very close to those from the Ru@C run. This could be explained by the similar properties between Ru@C and Ru@CNS-N_2_, such as Ru particle sizes, specific surface areas, and contents of Ru^0^ species, also reflected the inapplicability of N_2_ atmosphere in doping of heteroatoms into carbon materials to prepare catalyst. On the other hand, as discussed in Section 3.1, the CO_2_ atmosphere not only obviously improved the specific surface area but also helped the doping of sulfur into the carbon skeleton of char, thus leading to finer Ru particle size, more Ru^0^ content, and more weak acid sites. Unsurprisingly, the yield of total phenolic monomers significantly increased to 36.41 wt% in the case of Ru@CNS-CO_2_ (see Table 2), demonstrating the importance of CO_2_ for the doping process. This yield was even higher than the data from our previous study by a factor of 1.34, in which Ru supported on aminated carbon nanotubes was employed as the catalyst (Wu et al., 2021). The most abundant compound from the Ru@CNS-CO_2_ run was still compound **12** with a yield as high as 13.29 wt%, suggesting a promising hydrogenation ability of Ru@CNS-CO_2_. Different with the runs over Ru@C and Ru@CNS-N_2_, the second richest compound here was compound **10** (2,6-dimethoxy-4-propylphenol) rather than compounds **four** or **5**, implying that Ru@CNS-CO_2_ also had high activity in fragmentation of typical β-O-4 bonds (Wang et al., 2018; Wang et al., 2019). Based on the experimental results, a schematic diagram of lignin depolymerization to the main compounds can be proposed in Figure 7.

**TABLE 2:** Yields of phenolic monomers from catalytic depolymerization of lignin over different catalysts.

Entry	Name	Structure	Yield (wt%)			
			Blank	Ru@C	Ru@CNS-N_2_	Ru@CNS-CO_2_
1	Phenol	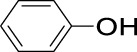	0.25	0.20	0.23	0.21
2	2-Methoxyphenol	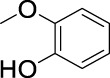	0.37	0.25	0.37	0.29
3	2-Methoxy-4-methylphenol	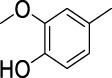	0.28	0.23	0.31	0.47
4	4-Ethylphenol	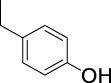	1.01	4.03	5.31	3.77
5	4-Ethyl-2-methoxyphenol	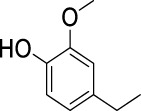	0.49	4.47	5.23	5.31
6	4-Vinylphenol	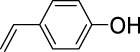	0.82	0.16	0.12	0.13
7	2-Methoxy-4-propylphenol	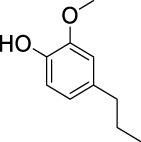	0	0.27	0.21	0.44
8	2,6-Dimethoxyphenol	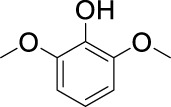	0.61	0.68	0.36	0.31
9	4-Ethyl-2,6-dimethoxyphenol	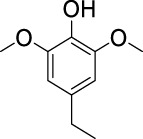	0.15	0.40	0.21	0.22
10	2,6-Dimethoxy-4-propylphenol	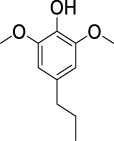	0.20	0.81	1.61	6.74
11	Methyl 3-(4-hydroxyphenyl)propanoate	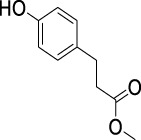	0.30	1.89	2.31	4.53
12	Methyl 3-(4-hydroxy-3-methoxyphenyl)propanoate	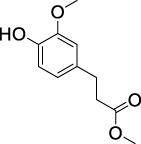	0.49	5.14	5.32	13.29
13	4-Allyl-2,6-dimethoxyphenol	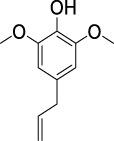	0.17	0.32	0.41	0.38
14	Methyl ferulate	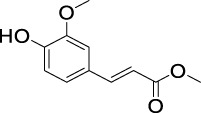	2.38	1.49	0.88	0.11
15	Methyl coumarate	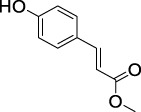	2.74	0.13	0.07	0.21
16	Total		10.25	20.47	22.95	36.41

## Conclusion

Ru supported on nitrogen and sulfur co-doped biochar catalysts were prepared for hydrogenolysis of technical lignin to phenolic monomers. The effect of atmosphere for the doping process on catalytic performance was studied by using N_2_ and CO_2_. The following conclusions can be drawn based on the experimental results:

1) The physicochemical structures of CNS-N_2_ and CNS-CO_2_ supports were significantly different. Compared with CNS-N_2_, CNS-CO_2_ was characterized by larger specific surface area, richer C=S and C-S groups, and higher oxygen content.
2) The unique structure of CNS-CO_2_ support can prevent the agglomeration of Ru particles. The particle size of Ru from Ru@CNS-CO_2_ was as small as 1.41 nm, which was significantly smaller than that from Ru@CNS-N_2_ (4.46 nm). Furthermore, the Ru^0^ species in Ru@CNS-CO_2_ as well as its acid sites were much richer than those in Ru@CNS-N_2_.
3) The yield of phenolic monomers from corncob-derived lignin hydrogenolysis over Ru@CNS-CO2 was as high as 36.41 wt%, which was higher than that over Ru@CNS-N_2_ by a factor of 1.6.

## Data Availability

The original contributions presented in the study are included in the article/Supplementary Material, further inquiries can be directed to the corresponding authors.
